# Thermally-curable nanocomposite printing for the scalable manufacturing of dielectric metasurfaces

**DOI:** 10.1038/s41378-022-00403-0

**Published:** 2022-07-04

**Authors:** Wonjoong Kim, Gwanho Yoon, Joohoon Kim, Heonyeong Jeong, Yeseul Kim, Hojung Choi, Trevon Badloe, Junsuk Rho, Heon Lee

**Affiliations:** 1 Department of Materials Science and Engineering, Korea University, Seoul, 02841 Republic of Korea; 2 Department of Mechanical Engineering, Pohang University of Science and Technology (POSTECH), Pohang, 37673 Republic of Korea; 3 Department of Manufacturing Systems and Design Engineering, Seoul National University of Science and Technology, Seoul, 01811 Republic of Korea; 4 Department of Chemical Engineering, Pohang University of Science and Technology (POSTECH), Pohang, 37673 Republic of Korea; 5 POSCO-POSTECH-RIST Convergence Research Center for Flat Optics and Metaphotonics, Pohang, 37673 Republic of Korea; 6 National Institute of Nanomaterials Technology (NINT), Pohang, 37673 Republic of Korea

**Keywords:** Nanophotonics and plasmonics, Structural properties, Micro-optics, Nanoparticles

## Abstract

Metasurfaces consisting of artificially designed meta-atoms have been popularized recently due to their advantages of amplitude and phase of light control. However, the electron beam lithography method for metasurface fabrication has high cost and low throughput, which results in a limitation for the fabrication of metasurfaces. In this study, nanocomposite printing technology is used to fabricate high-efficiency metasurfaces with low cost. To demonstrate the efficiency of the proposed fabrication method, a metahologram is designed and fabricated using a nanocomposite. The metahologram exhibits conversion efficiencies of 48% and 35% at wavelengths of 532 and 635 nm, respectively. The nanocomposite is composed of polymers with nanoparticles, so durability tests are also performed to evaluate the effects of temperature and humidity on the metasurfaces. The test verifies that at temperatures below the glass transition temperature of the base resin, the nanostructures do not collapse, so the efficiency of the metasurfaces remains almost the same. The surrounding humidity does not affect the nanostructures at all. Hence, the durability of the nanocomposite metasurfaces can be further enhanced by replacing the base resin, and this nanocomposite printing method will facilitate practical metasurface use at low cost.

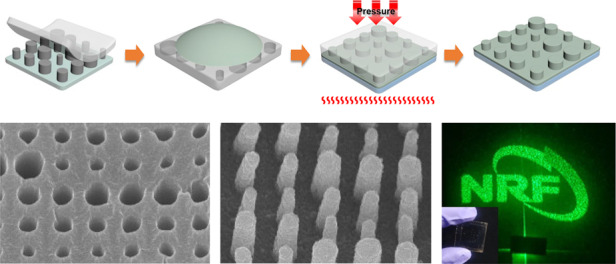

## Introduction

Metasurfaces consist of nanostructure arrays of subwavelength antennas that can allow control of electromagnetic waves. These nanoantennas can allow the control of optical properties according to their morphology, thickness, and material composition^1–6^. Therefore, metasurfaces exhibit novel optical effects and functions that cannot be easily achieved in nature^7–11^. Moreover, metasurfaces can replace complex optical systems with virtually flat formfactors. The special functionalities of metasurfaces have been applied in various fields, such as metalenses^12–17^, holographic devices^18–29^, optical cloaks^30,31^, and color filters^32–38^. Active metasurfaces have also been investigated extensively to facilitate dynamic light manipulation^39–42^, as substantial research has been conducted on machine learning-based inverse design methods^43–46^. However, for the practical application of metasurfaces, it is necessary to overcome various fabrication limitations, such as manufacturing cost and production efficiency.

The realization of optical metasurfaces requires precise lithography processes due to the necessity of using resolutions down to several hundreds of nanometers or less. The two main techniques used to fabricate metasurfaces, electron beam lithography (EBL) and focused ion beam milling, enable precise lithography but come with optical diffraction issues^47–49^. Since the total area of these techniques is very small and expensive procedures, such as vacuum deposition, are needed, new methods for fabricating practical metasurfaces are required^50,51^. Nanoimprint lithography (NIL) is a method that offers the advantage of the rapid and easy manufacturing of nanostructures using a solution-based process. The nanostructures are produced using a single direct contact with a replica mold^52–54^. This method is fast and cost-effective, as it does not require any vacuum processes^55^. Although the master stamp must be created using traditional EBL processes^56^, it can be used repeatedly. It can also be applied to various resins and incorporated with light sources of various wavelengths *λ* by creating a single stamp^57^.

In this study, we design and fabricate a metahologram using NIL. As the designed master stamp has a high aspect ratio, it is difficult to replicate its nanostructure with typical polydimethylsiloxane (PDMS); thus, the master stamp is replicated with hard PDMS (h-PDMS). In the replicated polymer mold, the pattern is transferred to the substrate through dielectric nanoparticle-embedded thermally curable polymer resin (PER), which has a sufficiently high refractive index for use in metasurface fabrication. Therefore, by simply duplicating the master stamp through the PER, a dielectric metahologram that forms a holographic image can be fabricated easily. This approach is inexpensive and highly productive compared to other metasurface manufacturing processes, as vacuum deposition and etching are not needed. The durability of the PER metasurfaces is investigated at different temperatures and relative humidities. The PER nanostructures do not collapse under temperatures below the glass transition temperature of the base resin, and the metasurface efficiency also remains constant. Furthermore, the relative humidity does not affect the PER nanostructures and the metasurface efficiency.

## Results

**Fig. 1:**
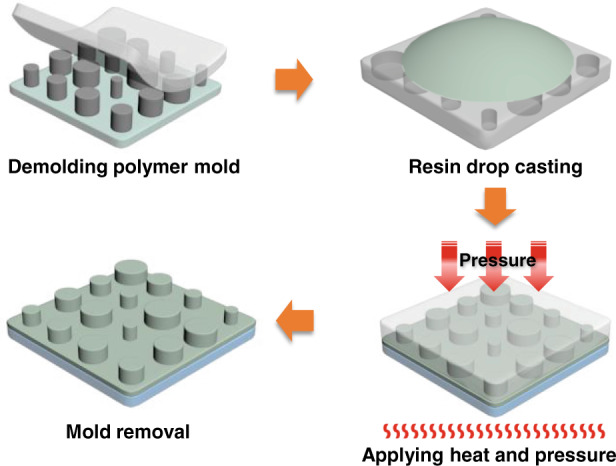
Schematic of metasurface fabrication by nanocomposite printing

Figure 1 presents a schematic of the fabrication of the metasurfaces using NIL. First, the h-PDMS mold is duplicated from the master stamp. As the single h-PDMS layer is brittle, it can easily be crushed; thus, an additional PDMS buffer layer is coated onto the h-PDMS thin film. Thereafter, the resin is dropped onto the replicated mold, which is covered with a substrate, the temperature is increased to 80 °C, and a pressure of 2 bar is applied for 20 min. The PER consists of TiO_2_ nanoparticles, dipentaerythritol hexaacrylate (DPHA), and tert-butyl peroxybenzoate (trigonox C). DPHA acts as a binder that supports the nanostructures by connecting the nanoparticles, while trigonox C promotes the action of DPHA as a thermal initiator. The excess solvent is absorbed into the polymer mold, resulting in the formation of PER nanostructures. In the process of transferring PER nanostructures to the substrate, the adhesive force of the PER on the substrate must be strong enough for easy demolding from the polymer mold. Once the polymer mold is removed, a PER metasurface is left on the substrate.

**Fig. 2:**
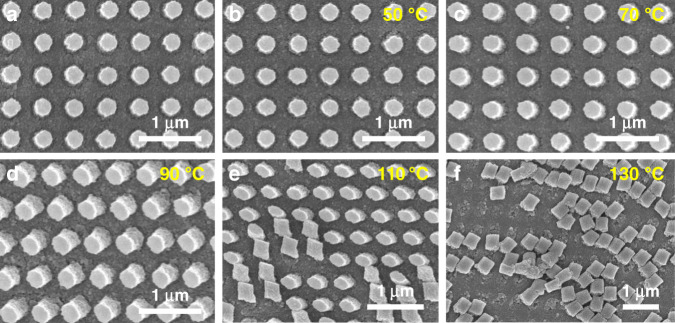
Effect of temperature on PER nanostructures. **a** Original PER nanostructures. **b**–**f** PER nanostructures after 4 h of annealing at different temperatures at a surrounding humidity of 30%. The PER patterns begin to collapse above 90 °C, which corresponds to the glass transition temperature of the base resin

**Fig. 3:**
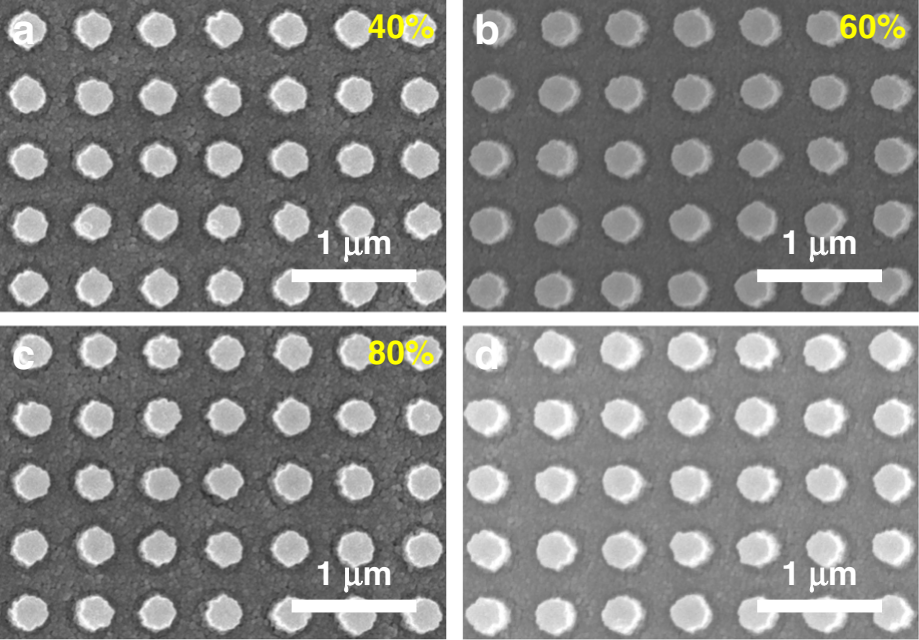
Effect of humidity on PER nanostructures. The test sample is identical to that of the temperature test. **a**–**c** PER nanostructures after 4 h in a humidity-controlled chamber. **d** PER nanostructures after soaking in water. The PER nanostructures are not affected by the surrounding humidity

The effects of temperature and humidity on the PER nanostructures are evaluated, as the base resin of the PER is a polymer that is sensitive to heat. A uniform array of similar cylindrical PER nanostructures constitutes the PER metasurfaces for the tests (Fig. 2a), and the deformation of the nanostructures is assessed after annealing for 4 h at different temperatures (Fig. 2b–f). The PER nanostructures begin to collapse when the temperature exceeds 90 °C, which is the glass transition temperature of DPHA. We believe that the thermal resistance of the PER could be enhanced by using other base resins with higher glass transition temperatures. The same metasurfaces are also used for humidity tests where the metasurface is exposed to different relative humidities in a humidity-controlled chamber (Fig. 3a–c). The humidity test verifies that the PER nanostructures are not affected by the surrounding humidity even if the sample is immersed in water (Fig. 3d).

**Fig. 4:**
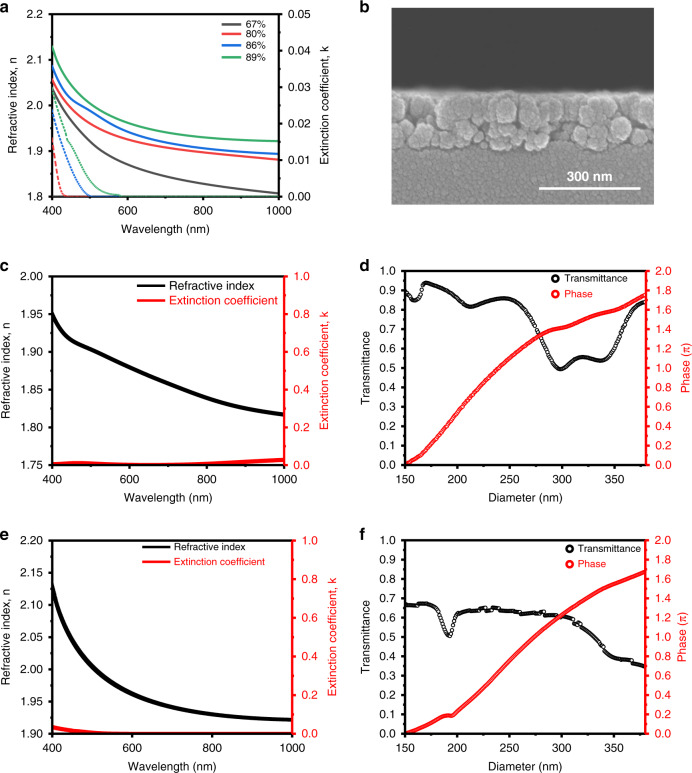
Optical properties of TiO_2_ PER thin films. **a** Refractive index (dashed lines) and extinction coefficient (solid lines) according to TiO_2_ nanoparticle concentration following solvent removal. **b** SEM image of a spin-coated 80% TiO_2_ nanoparticle layer on a glass substrate. Optical properties of TiO_2_ PER thin films: (**c**) measured refractive index and extinction coefficient and (**d**) simulated transmission of 80 wt% PER. The transmission is simulated at *λ* = 532 nm. **e** Refractive index and (**f**) simulated transmission of 89 wt% PER. The transmission is simulated at *λ* = 635 nm

It is required to arrange the nanostructures according to phase for the visualization of the hologram. To design appropriate nanostructures, the refractive index and extinction coefficient of the TiO_2_ PER are evaluated for various nanoparticle concentrations (Fig. 4a). A higher concentration results in a higher refractive index and extinction coefficient. By spin-coating and curing the PER, a uniform thin film is formed (Fig. 4b). The refractive index and extinction coefficient of the PER are measured using ellipsometry (Fig. S2). When the concentration of TiO_2_ PER is adjusted, 89 wt% PER shows a higher refractive index than 80 wt% PER, confirming that it is sufficiently applicable to other wavelength bands (Fig. 4c, d). A finite-difference time-domain (FDTD) simulation is performed based on the measured refractive index and extinction coefficient to optimize the hologram formation of the green and red lights. The height, period, and diameter of the cylindrical nanostructures are determined to achieve a transmission phase delay from 0 to 2π. The FDTD simulation reveals that 80 wt% TiO_2_ PER is suitable for green light and 89 wt% TiO_2_ PER is suitable for red light (Fig. 4e, f). When the diameter of the nanostructure is varied from 150 to 380 nm, the TiO_2_ PER can achieve the required phase shift for each *λ* by changing the concentration.

To design the metahologram, we use the Gerchberg-Saxton (GS) algorithm, which employs an iteration process to reduce the error between the desired and holographic images. The desired grayscale image is represented by a two-dimensional matrix consisting of the target intensity distribution intensities from 0 to 255. The complex amplitude profile in the object plane can be obtained by taking the inverse Fourier transform of the target image. Then, the magnitude of the transformed matrix is normalized by the GS algorithm so that the matrix contains phase information only. The Fourier transform of the consequent matrix represents the holographic image generated by a metasurface when it is encoded by the phase-only profile. However, the image has large errors compared with the desired image, so the magnitude of the image is normalized again and multiplied by the desired image. This process enforces the reduction of the error during the iteration process. After taking the inverse Fourier transform again, those processes are iterated. When the error becomes sufficiently small, the normalized phase-only matrix at the object plane is the phase profile required for a metasurface to produce the desired image.

**Fig. 5:**
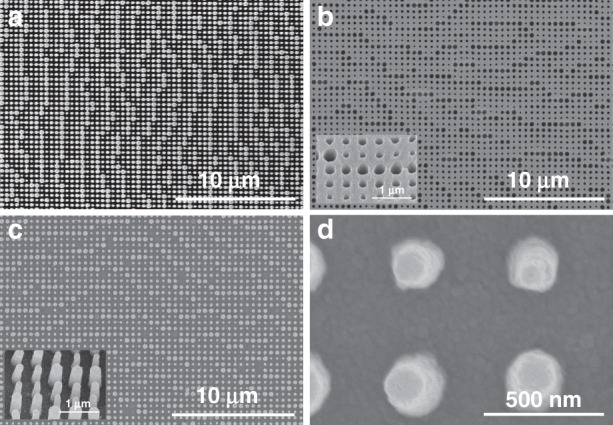
Scanning electron microscopy (SEM) images. **a** Si master stamp, **b** h-PDMS polymer replica. **c**, **d** TiO_2_ 80% nanoparticle PER nanostructures

The metasurface consists of eight types of cylindrical nanostructures with diameters that vary from 150 to 380 nm, with a periodicity of 500 nm and height of 800 nm (Fig. 5a). The polymer soft mold has a hole-shaped pattern that is the inverse of the master stamp. The SEM images of the h-PDMS verify that the master stamp is replicated effectively, without any loss of the desired pattern (Fig. 5b). Furthermore, the nanostructures of the master stamp are successfully replicated with the TiO_2_ PER (Fig. 5c, d). Although the pattern is composed of nanoparticles, it is uniform and smooth as a result of the small average diameter of the TiO_2_ nanoparticles of 27.3 nm, which is small compared to that of the pattern size (Fig. S1).

**Fig. 6:**
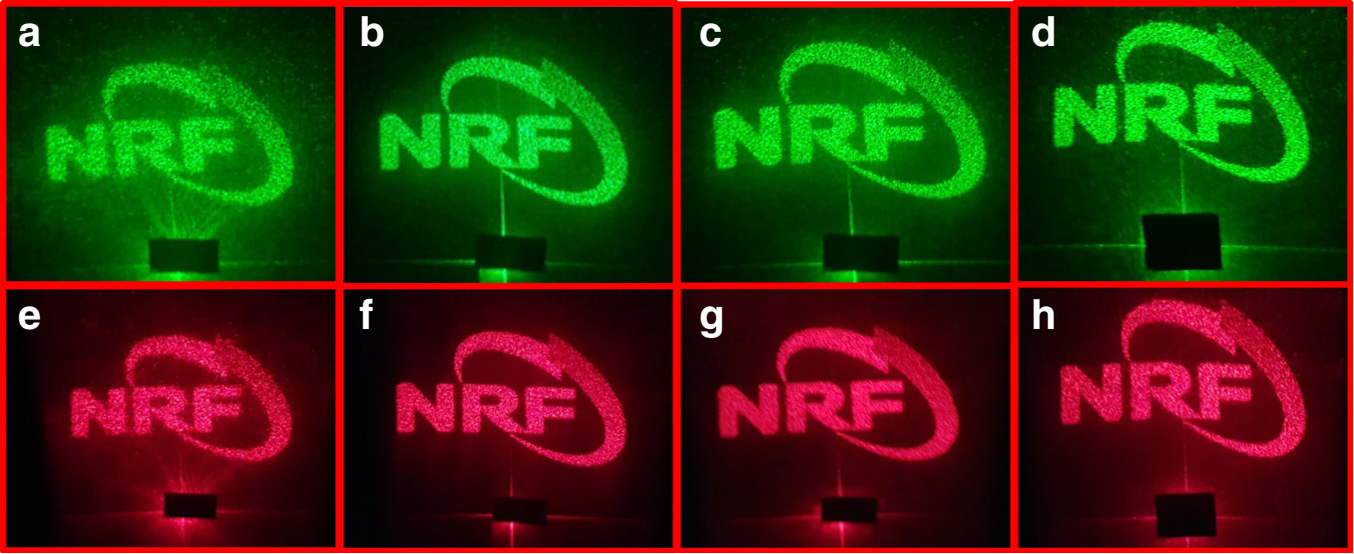
Captured holographic images produced by TiO_2_ PER metaholograms with different concentrations. Measurement results at *λ* = 532 nm with concentrations of (**a**) 67 wt%, (**b**) 80 wt%, (**c**) 86 wt%, and (**d**) 89 wt% and at *λ* = 635 nm with concentrations of (**e**) 67 wt%, (**f**) 80 wt%, (**g**) 86 wt%, and (**h**) 89 wt%. **i** Measured hologram efficiency according to TiO_2_ PER concentration

**Fig. 7:**
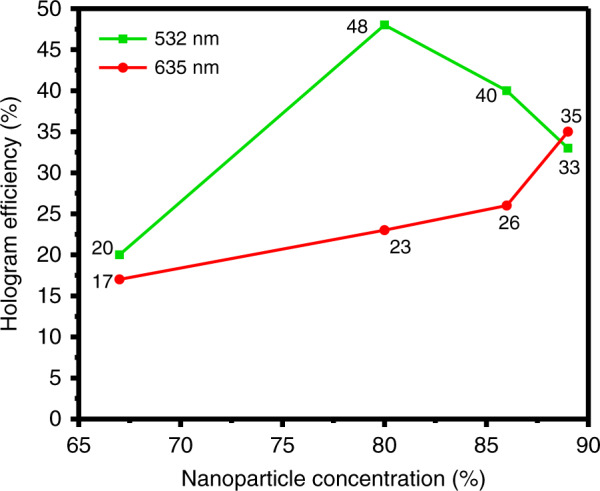
Measured hologram efficiency according to TiO_2_ PER concentration

The designed metahologram is fabricated using TiO_2_ PER, and the holographic image is observed by shining lasers of *λ* = 532 and 635 nm (Fig. 6). When *λ* = 532 nm, the 80 wt% PER yields the clearest image (Fig. 6b). When *λ* = 635 nm, the 89 wt% PER yields the clearest image (Fig. 6h). Figure 7 depicts the hologram efficiency, which is the optical power ratio of the incident light to the holographic image. The maximum efficiency is 48% at *λ* = 532 nm and 35% at *λ* = 635 nm. The FDTD simulation and experimental values are consistent, but the efficiency of the fabricated metahologram is lower than the FDTD simulation result because of fabrication defects. The transmission coefficients of the metasurfaces are affected by the physical shapes of the individual nanostructures, but the metasurfaces in this work have several kinds of defects, such as slanted sidewalls and random deformation (Fig. S3).

**Fig. 8:**
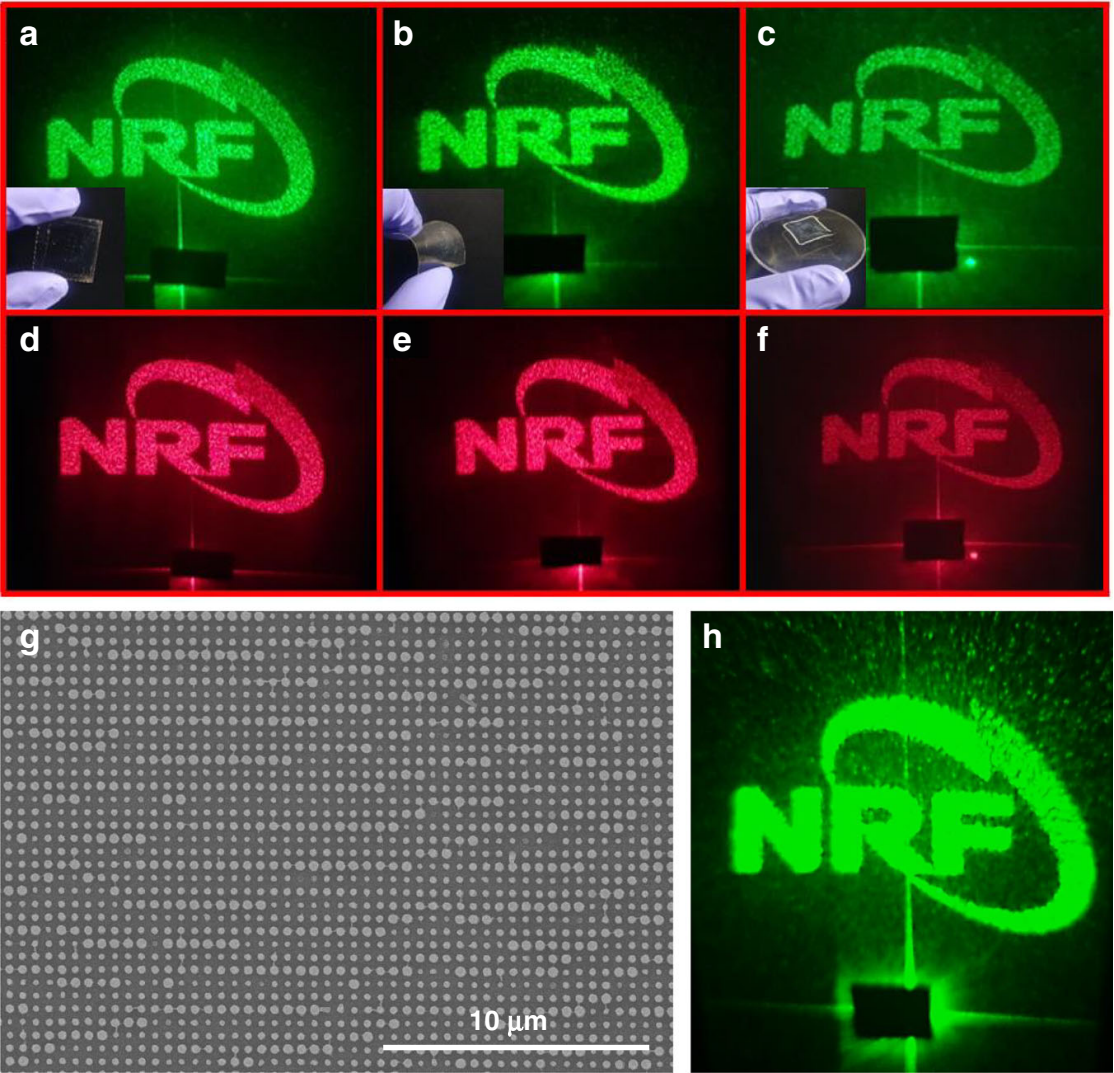
Captured holographic images generated from TiO_2_ PER metaholograms. They are fabricated on (**a**) and (**d**) flat, (**b**) and (**e**) flexible, and (**c**) and (**f**) curved substrates. Metaholograms following the durability test: (**g**) SEM image and (**h**) generated hologram after 24 h at 70 °C and 70% humidity

NIL is a solution-based and direct-contact type of lithography. As the replicated mold is flexible, patterns can easily be printed on various substrates. In addition to flat glass, the patterns can be formed on flexible and curved glass substrates (Fig. 8). Although the clarity is reduced owing to the phase deviation resulting from the substrate curvature, the hologram can be easily recognized. Moreover, less distortion of the structure occurs on substrates with higher curvatures than other lithography types. Therefore, our approach can be applied to create metasurfaces on curved substrates.

A reliability test is again conducted in a high-temperature and high-humidity environment to verify the durability of the PER metasurfaces that are produced through the solution-based NIL process. The metasurfaces are stored for 24 h in an atmosphere of 70% humidity at 70 °C. The SEM images confirm that the nanostructures are not deformed (Fig. 8g). The PER synthesized in this study was stable in high-temperature and high-humidity environments. Figure 8h depicts a holographic image following the reliability test. Moreover, the conversion efficiency of the metahologram is 43% after the test. No significant performance degradation occurs compared to the efficiency of 48% before the reliability test.

## Discussion

There have been various reported works on monolithic TiO_2_ metasurfaces, allowing for comparison. In the case of polarization-insensitive metasurfaces, a transmittance of 83% at a wavelength of 532 nm has been proven^58^. The efficiency of the monolithic metasurfaces is higher than that of TiO_2_ PER metasurfaces in this work because the refractive index of TiO_2_ is much higher than that of TiO_2_ PER. However, it is expensive and time-consuming to fabricate monolithic TiO_2_ metasurfaces because they require challenging processes, such as the atomic layer deposition of TiO_2_ over 500 nm. In contrast, our TiO_2_ PER metasurfaces can be rapidly fabricated at very low cost once a master mold is prepared. Moreover, TiO_2_ PER metasurfaces can be defined for any kind of substrate, including flexible polymers and curved glass, which are not compatible with typical monolithic TiO_2_ metasurfaces.

In this study, although it is necessary to produce a master stamp using electron beam lithography, a high-efficiency metahologram is produced through a semipermanently usable master stamp. By producing reusable polymer molds using h-PDMS with excellent mechanical strength, metasurfaces can be replicated repeatedly with a low cost. The TiO_2_ PER, which has a high refractive index, is synthesized and applied in the production of metaholograms. Furthermore, the concentration is optimized to control the refractive index of the PER. Using the solution-based NIL process, it is possible to create metasurfaces on flexible and curved substrates. The reliability test validates that the PER metasurfaces maintain their optical performance at temperatures below the glass transition temperature of the base resin. The surrounding humidity does not influence the PER metasurface. As high fabrication cost is a major limitation in the metasurface field, this low-cost and high-efficiency NIL-based manufacturing method may provide a breakthrough in the commercialization of metasurfaces.

## Supplementary information

Supplementary Information
