# Supplementary material for: Thermally-curable nanocomposite printing for the scalable manufacturing of dielectric metasurfaces
Source: Microsyst Nanoeng. 2022 Jul 4;8:73. doi: 10.1038/s41378-022-00403-0 (PMC9253025; doi:10.1038/s41378-022-00403-0)
Supplement: Supplementary file 1 — Supplementary Information [file 41378_2022_403_MOESM1_ESM.pdf]

Supplementary Information

Thermally-curable nanocomposite printing for scalable manufacturing of dielectric metasurfaces

Wonjoong Kim[1,†], *Gwanho Yoon*[2,3,†], *Joohoon Kim*[2,†], *Heonyeong Jeong*[2], *Yeseul Kim*[2], *Hojung Choi*[1], *Junsuk Rho*[2,4,5,6,*], *and Heon Lee*[1,*]

[1]*Department of Materials Science and Engineering, Korea University, Seoul 02841, Republic of Korea*

[2]*Department of Mechanical Engineering, Pohang University of Science and Technology (POSTECH), Pohang 37673, Republic of Korea*

[3]*Department of Manufacturing Systems and Design Engineering, Seoul National University of Science and Technology, Seoul 01811, Republic of Korea*

[4]*Department of Chemical Engineering, Pohang University of Science and Technology (POSTECH), Pohang 37673, Republic of Korea*

[5]*POSCO-POSTECH-RIST Convergence Research Center for Flat Optics and Metaphotonics, Pohang 37673, Republic of Korea*

[6]*National Institute of Nanomaterials Technology (NINT), Pohang 37673, Republic of Korea*

*Correspondence: Junsuk Rho (jsrho@postech.ac.kr), Heon Lee (heonlee@korea.ac.kr)

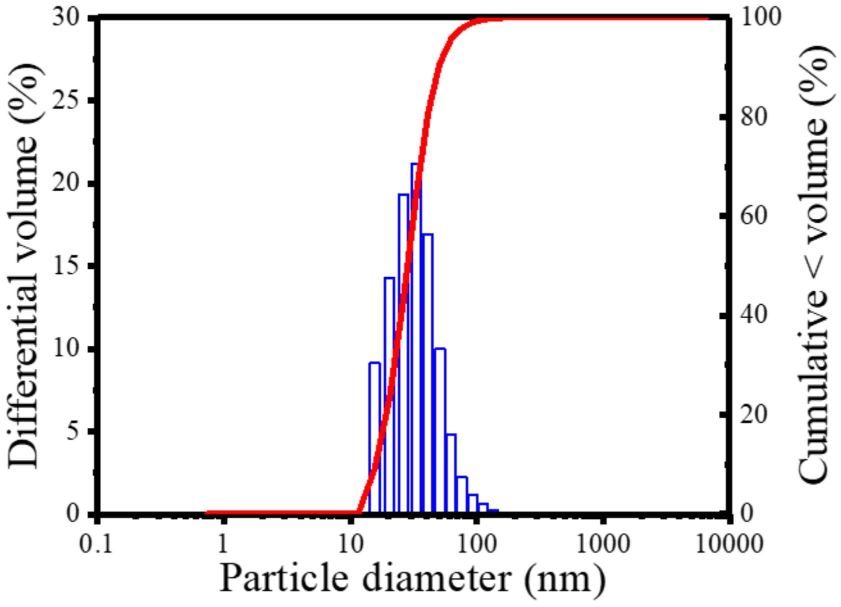

Fig. S1 Size distribution of TiO_2 nanoparticles in TiO_2 PER. The size of TiO_2 nanoparticles is measured to apply PER to the nanostructure fabrication. The average diameter of the TiO_2 nanoparticles is 27.3 nm, which is suitable for metasurfaces with a minimum line width of 150 nm.

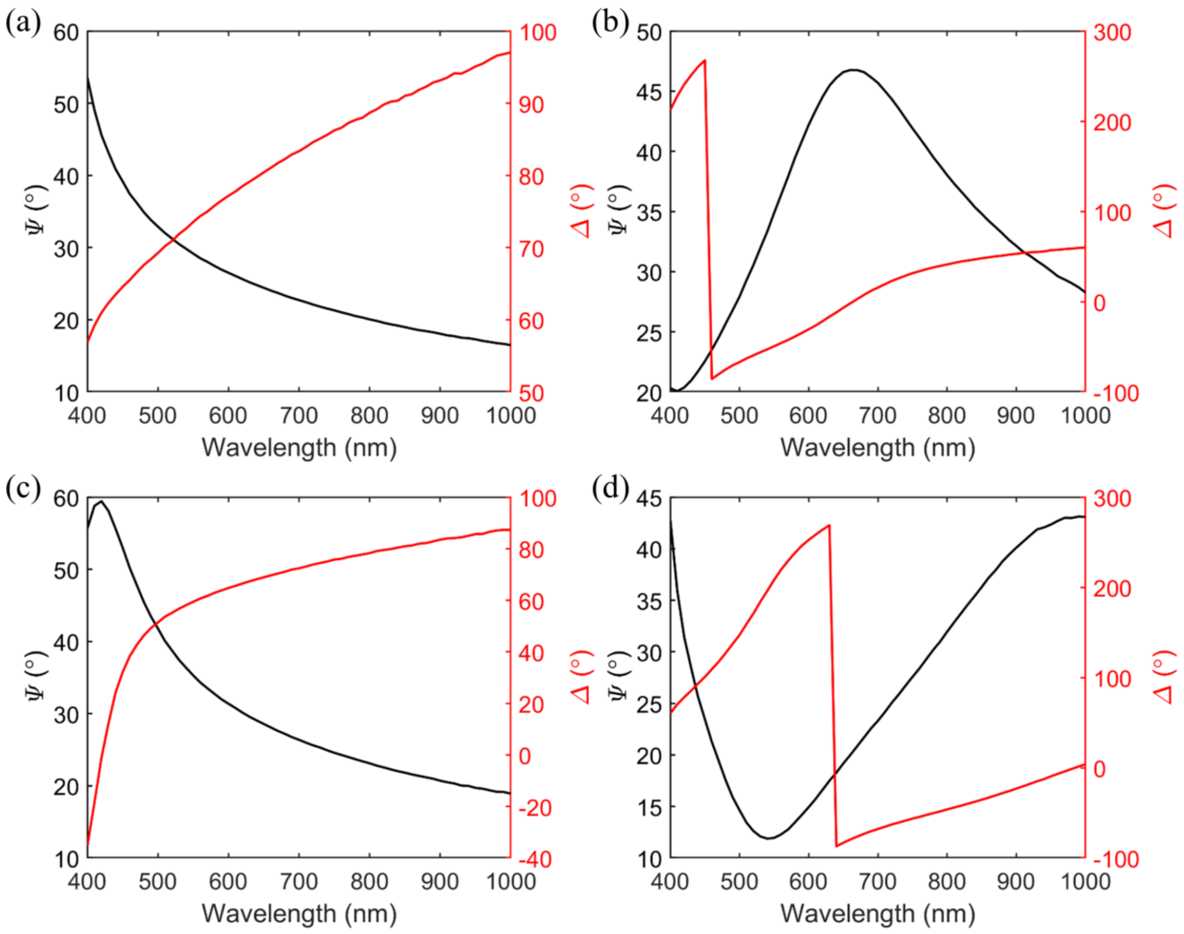

Fig. S2 Measured ellipsometry results of the fabricated TiO_2 PER. Nanoparticle concentration: (a) 67 wt%; (b) 80 wt%; (c) 86 wt%; (d) 89 wt%.

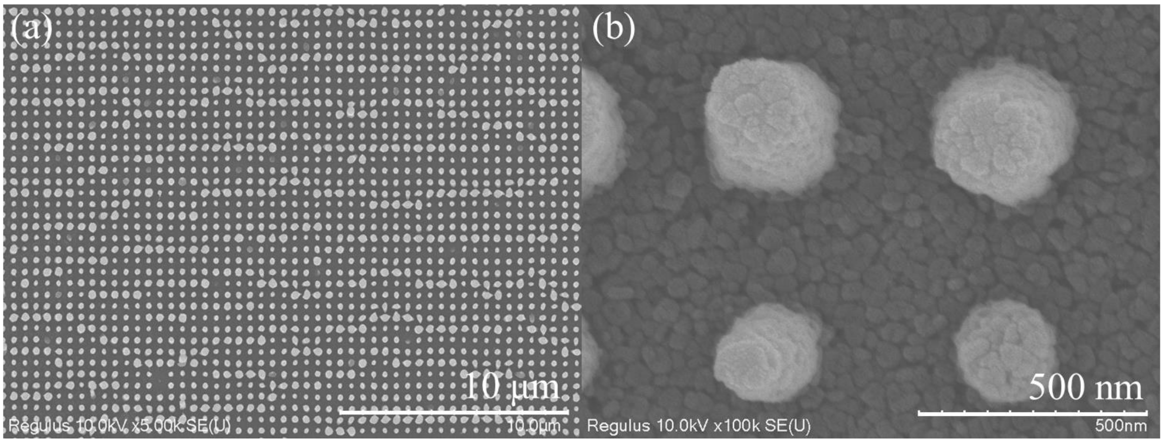

Fig. S3 Characterization of the fabricated PER metasurfaces. (a) SEM image of TiO_2 89% nanoparticle PER nanostructures in metasurfaces. Those nanoparticles have the same diameter as the TiO_2 80% nanoparticle PER. The nanostructures are uniformly formed over a large area. (b) The magnified SEM image.
